# Prognostic significance of bone marrow FDG uptake in patients with gynecological cancer

**DOI:** 10.1038/s41598-021-81298-1

**Published:** 2021-01-26

**Authors:** Kotaro Shimura, Seiji Mabuchi, Naoko Komura, Eriko Yokoi, Katsumi Kozasa, Tomoyuki Sasano, Mahiru Kawano, Yuri Matsumoto, Tadashi Watabe, Michiko Kodama, Kae Hashimoto, Kenjiro Sawada, Jun Hatazawa, Tadashi Kimura

**Affiliations:** 1 Department of Obstetrics and Gynecology, Osaka University Graduate School of Medicine, 2-2 Yamadaoka, Suita, Osaka 565-0871 Japan; 2 Department of Obstetrics and Gynecology, Nara Medical University, Kashihara, Nara 634-8522 Japan; 3 Department of Gynecologic Oncology and Reproductive Medicine, The University of Texas MD Anderson Cancer Center, Houston, 77030 USA; 4 Department of Nuclear Medicine and Tracer Kinetics, Osaka University Graduate School of Medicine, 2-2 Yamadaoka, Suita, Osaka 565-0871 Japan

**Keywords:** Cancer imaging, Gynaecological cancer

## Abstract

We investigated the prognostic significance and the underlying mechanism of increased bone marrow (BM) 2-(^18^F) fluoro-2-deoxy-D-glucose as a tracer (FDG)-uptake in patients with gynecological cancer. A list of patients diagnosed with cervical, endometrial, and ovarian cancer from January 2008 to December 2014 were identified. Then, through chart reviews, 559 patients who underwent staging by FDG-positron emission tomography (PET)/computed tomography (CT) and subsequent surgical resection were identified, and their clinical data were reviewed retrospectively. BM FDG-uptake was evaluated using maximum standardized uptake value (SUVmax) and BM-to-aorta uptake ratio (BAR). As a result, we have found that increased BAR was observed in 20 (8.7%), 21 (13.0%), 21 (12.6%) of cervical, endometrial, and ovarian cancer, respectively, and was associated with significantly shorter survival. Increased BAR was also closely associated with increased granulopoiesis. In vitro and in *vivo* experiments revealed that tumor-derived granulocyte colony-stimulating factor (G-CSF) was involved in the underlying causative mechanism of increased BM FDG-uptake, and that immune suppression mediated by G-CSF-induced myeloid-derived suppressor cells (MDSCs) is responsible for the poor prognosis of this type of cancer. In conclusion, increased BM FDG-uptake, as represented by increased BAR, is an indicator of poor prognosis in patients with gynecological cancer.

## Introduction

Positron emission tomography (PET) with 2-(^18^F) fluoro-2-deoxy-D-glucose as a tracer (FDG PET) is a functional diagnostic technique based on the rationale that rapidly dividing malignant cells have increased glucose metabolism, allowing the detection of areas with cancer cells. To overcome the inherent disadvantages of FDG-PET scanning (i.e. poor anatomical information), integrated FDG-PET/computed tomography (CT) has been developed and is widely used for staging, determining the extent of surgical resection, or planning radiation fields in the management of gynecological cancers^1–3^. Moreover, recent clinical studies have suggested that FDG-uptake in a primary tumor can serve as an indicator of treatment response or survival outcomes.

Bone marrow (BM) is a key component of the hematopoietic and lymphatic system. It is known that ^18^F-FDG accumulates physiologically in BM, reflecting its hematopoietic activity. Although BM FDG-uptake in patients with cancer is generally moderate, we sometimes encountered patients showing relatively high BM FDG-uptake during pretreatment workup. According to previous studies, BM FDG-uptake is associated with serum C-reactive protein level, transforming growth factor-beta level, white blood cell count, and neutrophil count^4–6^. Thus, BM FDG-uptake in patients with cancer is believed to reflect the degree of systemic inflammatory response to a malignant tumor.

Recently, systemic inflammatory responses including leukocytosis, neutrophilia, or increased neutrophil to lymphocyte ratio (NLR) have gained attention as indicators of poor prognosis in patients with various solid malignancies^7–10^. Thus, pretreatment BM FDG-uptake can serve as a useful prognostic indicator in patients with gynecological cancer. However, the clinical significance of increased BM FDG-uptake in patients with gynecological cancer as well as the underlying mechanism of increased BM FDG-uptake in relation to patient’s prognosis remain largely unknown.

In the current study, using clinical data obtained from in patients with gynecological cancer, we first evaluated the prognostic significance of increased BM FDG-uptake. Then, using tumor samples obtained from these patients, as well as the animal models of gynecological cancers, we performed mechanistic investigations focusing on tumor-derived granulocyte colony-stimulating factor (G-CSF), G-CSF-mediated hematopoietic activity, and myeloid-derived suppressor cells (MDSCs).

## Materials and methods

### Patients and clinical samples

This study was approved by the Osaka University Hospital’s Institutional Review Board (IRB). The analysis of the patient-derived data and all experiments were carried out in accordance with the Declaration of Helsinki. A list of patients who had newly diagnosed cervical, endometrial, or ovarian cancer at Osaka University Hospital from January 2008 to December 2014 were identified. Then, through chart reviews, patients who underwent staging FDG-PET/CT and subsequent surgical resection were identified. Patients who: (1) had a distant metastasis, (2) received neoadjuvant treatment, (3) had a history of another malignancy, (4) had concurrent infectious disease, or (5) had received erythropoietin, G-CSF, or granulocyte–macrophage colony-stimulating factor within 1 year were excluded. Clinical information regarding demographic or pathologic data, oncological and surgical outcome, as well as imaging results were collected from medical record and retrospectively analyzed. Cervical tumor tissue and blood samples were also collected and archived according to protocols approved by the IRB of Osaka University Hospital. Appropriate informed consent for the retrospective investigation was obtained from each patient.

### PET/CT protocol

Informed consent was obtained from each patient for FDG-PET/CT scanning. The FDG-PET/CT scans were obtained within 4 weeks of surgery. No patients received neoadjuvant chemotherapy before surgery. Whole-body imaging using ^18^F-FDG was carried out with a combined PET/CT scanner (Gemini GXL 16; Philips, Amsterdam, The Netherlands, or SET-3000 GCT/X; Shimadzu, Kyoto, Japan), which provide separate CT and PET datasets that can be accurately fused on a workstation (Voxbase; J-MAC System, Inc, Japan), as reported previously^11^. All patients fasted for at least 4 h prior to the intravenous administration of ^18^F-FDG at a dose of 3.7 MBq/kg. Whole-body images, generally from the top of the skull to mid thigh, were acquired in the supine position about 60 min after ^18^F-FDG injection. Image acquisition was initiated with a CT scan for attenuation correction and anatomical localization. The CT scanning parameters were as follows: 120 kV; 60–80 mA; 16 slices; 1.5 mm detector collimation; and 5.0 mm slice thickness. A whole-body emission PET scan was performed immediately after the CT scan in three-dimensional mode with a 3.0 min per bed position (11 positions), using a dedicated scanner with 32 rings of bismuth germanate detectors that simultaneously produced 63 slices of 3.125 mm thickness along a 20 cm longitudinal field. Attenuation-corrected PET images were reconstructed by row-action maximum likelihood algorithm method (RAMLA) in Gemini GXL 16 and dynamic row-action maximum likelihood algorithm (DRAMA) method in SET-3000 GCT/X, respectively.

### Image analysis

The CT and PET images were transferred to a commercially available workstation (Advantage Windows Workstation, version 4.5; GE Healthcare). For semiquantitative analysis of FDG-uptake, ROIs were defined on the target lesions (primary lesion and pelvic or paraaortic lymph nodes) on the transaxial PET images.

For the semi-quantitative analysis, the standardized uptake value (SUV) was calculated as the ratio of the image derived radioactivity concentration to the whole body radioactivity concentration. Physical decay (109.7 min) was corrected to the time of injection. The maximum standardized uptake value (SUVmax) within the regions of interest (ROI) was calculated as follows: concentration of radioactivity in the VOI (MBq/mL) × total body weight (kg)/injected radioactivity (g/MBq).

All FDG-PET/CT images were retrospectively interpreted by a gynecologist (K.S., with 8 years’ experience in gynecology) with the consensus of an experienced radiologist (T.W., with 12 years of experience in oncologic PET) who had no knowledge of the other imaging results or the clinical data.

**Figure 1:**
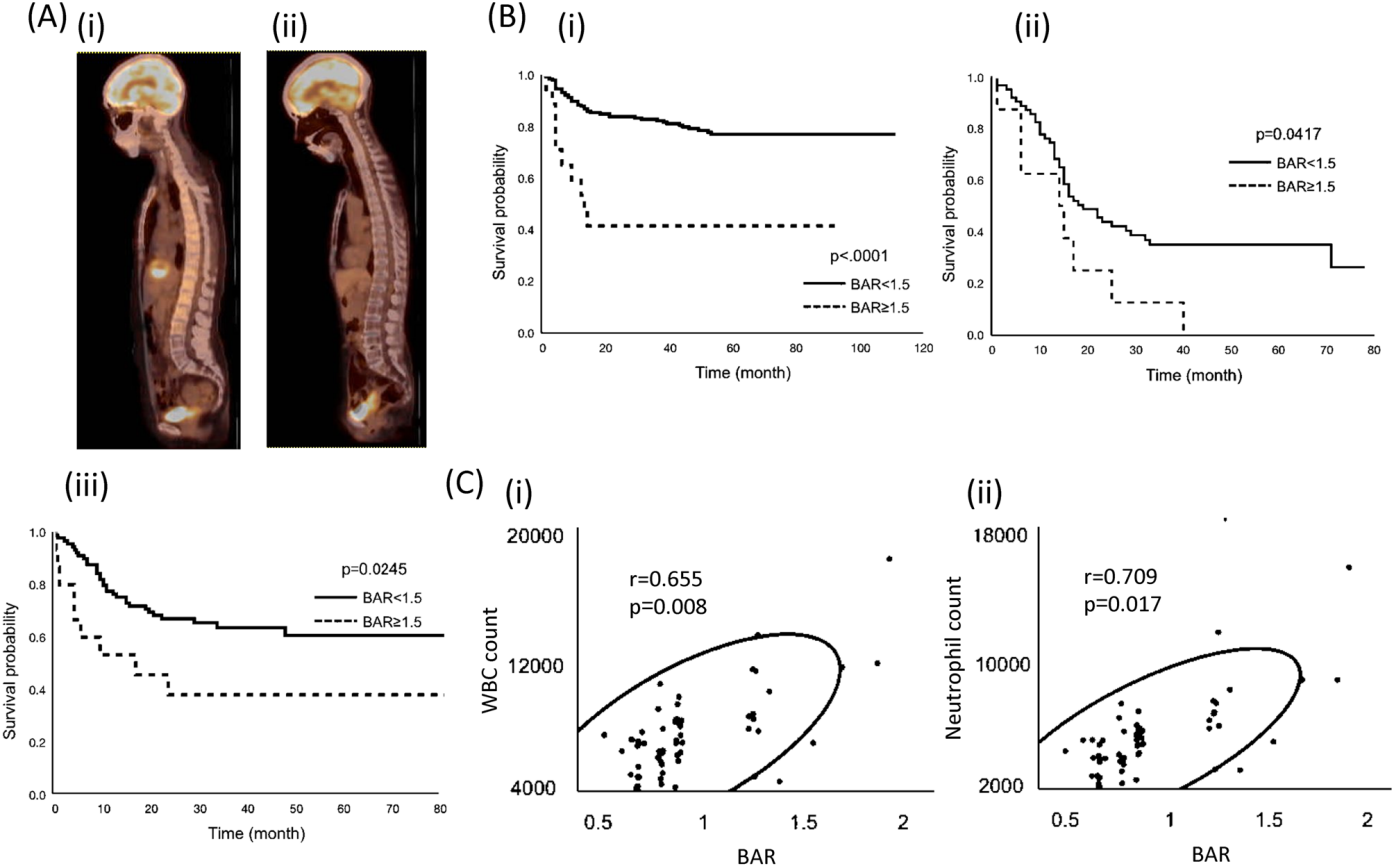
Clinical implications of BM FDG-uptake in patients with gynecologic cancer. (**A**) Fused sagittal 2-(^18^F) fluoro-2-deoxy-D-glucose as a tracer (FDG)-positron emission tomography/computed tomography (PET/CT) images. (i), patients with diffuse bone marrow FDG-uptake. (ii), patients with average bone marrow (BM) FDG-uptake. (**B**) Significance of BAR in patients with cervical cancer, patients with advanced stage ovarian cancer and patients with endometrial cancer. (i) Kaplan–Meier estimates of progression-free survival in patients with cervical cancer (higher-BAR group vs. lower-BAR group n = 231). (ii) Kaplan–Meier estimates of progression-free survival in patients with advanced stage ovarian cancer (higher-BAR group vs. lower-BAR group n = 87). (iii) Kaplan–Meier estimates of progression-free survival in patients with advanced stage endometrial cancer (higher-BAR group vs. lower-BAR group n = 113). (**C**) Correlation between BAR and neutrophils in patients with cervical cancer. The black circle; a density ellipse indicating correlation with a 95% confidence interval.

BM FDG-uptake varies between patients; some showed relatively high FDG-uptake and others show moderate FDG-uptake (Fig. 1A). To measure FDG-uptake of the BM, an ROI was drawn over the vertebral body of each of 5 vertebrae (Th8–Th12, unless a pathologic condition such as compression fracture or severe osteoarthritic changes was present). Mean SUV of the 5 selected vertebrae was calculated and defined as BM SUV. Afterwards, mean SUV of the 1 cm ROIs in the intra-aorta area was measured. Using the mean SUV of BM and intra-aorta area, the BM-to-aorta uptake ratio of FDG-uptake (BAR) was calculated (Supplemental Fig. 1).

### Reagents/antibodies

The following labeled monoclonal antibodies were used for the staining experiments: anti-human antibodies: V450-conjugated anti-CD33 and CD8 (eBiosciences, San Diego, CA), APC-conjugated anti-HLA-DR (Biolegend, San Diego, CA); anti-human/mouse antibodies: FITC-conjugated anti-CD11b (Tonbo Biosciences, San Diego, CA); anti-mouse antibodies: PE-conjugated anti-Gr-1 (R&D systems, Minneapolis, MN). A neutralizing antibody against Gr-1 (RB6-8C5) was purchased from R&D systems, Minneapolis, MN. The utility of these antibodies had been evaluated in our previous investigations^12^.

### Cell culture

ME180 cells (human cervical cancer), Ishikawa cells (human endometrial cancer), and ID-8 cells (mouse ovarian cancer) were purchased from the American Type Culture Collection. The cell lines were passaged in our laboratory soon after they were received from the cell bank, before being divided and stored in liquid nitrogen vessels. Each experiment was carried out using thawed cells without further authentication. The cells were maintained in Dulbecco’s modified Eagle’s medium (DMEM) supplemented with 10% fetal calf serum, as reported previously^12^.

### Clone selection

The expression vector for the mouse G-CSF gene (pCAmG-CSF) and the empty vector (pCAZ 2) used in this study, which were described previously, were provided by RIKEN BRC through the National Bio-Resource Project of MEXT, Japan. The expression of these genes was driven by the CAG promoter, as reported previously^13,14^. Transfection was performed using Lipofectamine 2000 (Invitrogen, Carlsbad, CA) in accordance with the manufacturer’s instructions. Clonal selection was performed by adding G-418 to the medium at a final concentration of 500 μg/mL, as reported previously^15^. ME-180 human cervical cancer cells stably transfected with the G-CSF expression vector or the empty vector were designated as ME-180-G-CSF and ME-180-Control, respectively. Ishikawa human endometrial cancer cells stably transfected with G-CSF expression vector or the empty vector were designated as Ishikawa-G-CSF and Ishikawa-Control, respectively. ID-8 mouse ovarian cancer cells stably transfected with G-CSF expression vector or the empty vector were designated as ID-8-G-CSF and ID-8-Control, respectively.

### Determination of G-CSF levels

The G-CSF concentrations of the serum samples were determined by enzyme-linked immunosorbent assay (ELISA) using Quantikine assay system for human G-CSF (R&D Systems, Minneapolis, MN) in accordance with the manufacturer’s protocol, as reported previously^12^.

### Real-time RT-PCR.

Real-time RT-PCR was performed using SYBR Green PCR master mix (Applied Biosystems, Carlsbad, CA) on a Step One Plus sequence detection system (Applied Biosystems, Carlsbad, CA), as reported previously^12^. Relative mRNA expression fold values were determined using standard deltaCt calculations. The PCR primers for GAPDH were purchased from Invitrogen (Carlsbad, CA), and those for Bv8 were purchased from Eurofins Operon (Huntsville, AL). The sequences of the primers used were as follows: GAPDH: forward primer, 5′-CCCTCAAGATTGTCAGCAATGC-3′ and reverse primer, 5′-GTCCTCAGTGTAGCCCAGGAT-3′.

### Immunohistochemistry

Tumor samples obtained from newly-diagnosed cervical cancer patients were fixed in 10% neutral buffered formalin, embedded in paraffin, sectioned, and processed for immunohistochemical staining. The primary antibody used were anti-CD33 and anti-CD8 antibody (eBiosciences, San Diego, CA). Optical image capture was performed using PROVIS AX80 (Olympus, Tokyo, Japan). The slides were examined using a bright field microscope by observer who were blinded to the patients’ clinical data, and counted the stained cells.

### In vivo tumor studies

All procedures involving animals and their care were approved by the animal care and usage committee of Osaka University, in accordance with institutional and NIH guidelines. The first experiment was conducted to examine the effect of G-CSF on survival. ME-180-control or ME-180-G-CSF cells, ID-8-control, or ID-8-G-CSF cells (5 × 10^6^ in 100 μL of phosphate-buffered saline [PBS]) were subcutaneously inoculated into 5–7-week-old C57BL/6 mice. Mice were observed every day and their survival was evaluated until death. If a tumor impaired the mobility of an animal, became ulcerated, or appeared infected, the mouse was euthanized. The second experiment was conducted to investigate the impact of tumor-derived G-CSF on BM FDG-uptake in the animal model of G-CSF-expressing gynecological cancer. Briefly, 7–8-week-old female F344/NJcl-rnu/rnu rats (NIH-RNU; Japan SLC, Shizuoka, Japan) were subcutaneously inoculated with either 2 × 10^7^ ME180-control, or ME180-G-CSF, Ishikawa-control, or Ishikawa-G-CSF cells in 200 μL of PBS into their right flanks. Three or 4 weeks after inoculation, the rats were anesthetized by intraperitoneal injection of a mixture with medetomidine, midazolam, and butorphanol, or inhalation of 2% isoflurane) and ^18^F-FDG (39.0–60.6 MBq/rat) was injected into the tail vein. Sixty minutes after injection, PET measurements were performed in an abdominal position using a small-animal FDG-PET/CT scanner (Inveon MM; Siemens Medical Solutions, Knoxville, USA). The image data acquired from the small-animal FGD-PET/CT scanner were displayed and analyzed with RadiAnt DICOM 4.6.9 Viewer (Medixant, Poznan—Poland), as reported previously^16^.

### Isolation of MDSCs

MDSCs were isolated from single-cell preparations of mouse splenocytes using an MDSC isolation kit (mouse) and an MS column (Miltenyi Biotec, Auburn, CA) in accordance with the manufacturer’s instructions. The purity of the isolated cell populations was determined by flow cytometry, and the frequency of CD11b^+^ Gr-1^+^ cells was > 95%^14^.

### Flow cytometry

Flow cytometric analysis was conducted as previously reported^12^. Briefly, single-cell suspensions were prepared from mouse blood, and tumor specimens. Red blood cells (RBCs) were removed using ammonium chloride lysis buffer. To prepare human samples, tumor cells and blood cells were filtered through 40 μm nylon strainers, incubated with antibodies, and analyzed by flow cytometry. Flow cytometric data were acquired using a FACSCanto II flow cytometer and analyzed using FACSDiva software (BD Biosciences, San Jose, CA). Cells that had been incubated with irrelevant isotype-matched antibodies and unstained cells served as controls.

### T cell proliferation assay

T cell proliferation assays were conducted as reported previously^12^. Briefly, a 96-well plate was coated with 1 μg/well of anti-CD3e antibody (Tonbo Biosciences, San Diego, CA). CD8-positive T cells were purified from the spleen of Balb/c mice using T cell isolation columns (R&D systems, Minneapolis, MN) in accordance with the manufacturer’s instructions. To determine the impact of MDSCs on T cell proliferation, purified MDSCs from the spleen of a G-CSF-treated mouse were co-cultured with T cells. Cell proliferation was assessed using a cell proliferation ELISA BrdU kit (Roche Applied Science, Penzberg, Germany).

### Statistical analysis

Continuous data were compared between groups the Student’s t test or Wilcoxon rank sum test. Frequency counts and proportions were compared between groups using chi-square test or a two-tailed Fisher’s exact test, as appropriate. A cut-off value for predicting progression was determined using ROC analysis in consideration of clinical usefulness for reads and diagnoses using an FDG-PET/CT image (Supplemental Fig. 2). As a result, the optimal cut-off values of BM SUVmax or BAR for predicting progression were defined as 1.53 or 1.50, respectively. Spearman rank correlation coefficients were used to assess the association between BAR and hematologic parameters. Correlation coefficient greater than 0.7 is considered strong correlation. Progression-free survival (PFS) was defined as the duration of the period from the day of inoculation to the detection of tumor progression or death from any cause. Moue survival was defined as the time from the day of inoculation to the date of death from any cause. We compared Kaplan–Meier curves for each subgroup using the log rank test. Cox proportional hazards regression analysis with stepwise variable selection was performed to identify significant independent predictors of overall survival (OS). p-values < 0.05 were considered statistically significant. All analyses were performed with JMP pro version 13.0.0 for Macintosh (SAS Institute Inc., Cary, NC).

## Results

### Patients

A total of 559 patients with gynecological cancers were included in this analysis (231 cervical, 167 endometrial, and 161 ovarian cancers). All patients underwent staging using FDG PET/CT before surgical resection. The median follow-up periods of patients with cervical, endometrial, and ovarian cancer were 55, 42.5, and 46 months, respectively.

### Prognostic significance of bone marrow SUVmax in patients with gynecological cancer

We first investigated the prognostic impact of increased SUV in the BM of gynecological cancer patients. As shown (Supplemental Tables 1–3), increased BM SUVmax (SUVmax > 1.53) was observed in 132 (57.1%), 84 (52.1%), and 82 (49.1%) patients with cervical, endometrial, and ovarian cancer, respectively. In the survival analyses using the Kaplan–Meier method and the univariate and multivariate Cox regression analyses, increased BM SUVmax was not found to be a significant prognosticator (Supplemental Fig. 3 and Supplemental Tables 4–6).

### Prognostic significance of BAR in patients with gynecological cancer

To further investigate the significance of BM FDG-uptake, we next employed a new marker BAR in which the ratio of mean SUV of BM and intra-aorta area was taken. As shown (Table 1, Supplemental Tables 7–8), increased BM FDG-uptake represented by increased BAR (BAR > 1.5) was observed in 20 (8.7%), 21 (13.0%) and 21 (12.6%) patients with cervical, endometrial, and ovarian cancer, respectively. When patients in the higher-BAR group were compared with those in the lower-BAR group, the higher-BAR group presented at more advanced clinical stages (in cervical and ovarian cancer), increased FDG-uptake in the lymph nodes (cervical and endometrial cancer), and had a lower hemoglobin levels (in cervical and endometrial cancer).

**Table 1:** Clinical characteristics of the lower- and higher-BAR group patients with cervical cancer.

	Lower-BAR group (n = 211)	Higher-BAR group (n = 20)	p-value
Age (y.o.)	56 (26–96)	48 (27–84)	0.477
FIGO stage			
I	97 (46)	3 (15)	< .0001
II	72 (34)	3 (15)	
III	24 (11)	2 (10)	
IV	18 (9)	12 (60)	
Primary treatment			
Surgery	113 (54)	6 (30)	0.021
Radiotherapy	96 (45)	14 (70)	
Others	2 (1)	0 (0)	
Histology			
SCC	148 (70)	13 (65)	0.827
Adeno	53 (25)	4 (20)	
Others	10 (5)	3 (15)	
Lymph node metastasis*			
Positive	37 (18)	10 (50)	0.002
Negative	174 (82)	10 (50)	
Tumor size (cm) **	35 (5–85)	50 (5–100)	0.212
Hemoglobin (g/dl)	12.7 (7.2–15.3)	9.4 (6.3–11.2)	0.037
CRP (mg/l)	0.04 (0–13.6)	0.37 (0–18.1)	0.123
BM SUV	1.69 (0.56–2,66)	2.22 (1.39–2.8)	0.003
BM/Ao ratio	1.09 (0.42–1.49)	1.55 (1.50–2.3)	< .0001

BAR, BM-to-Aorta ratio of FDG uptake; FIGO, International Federation of Gynecology and Obstetrics; SCC, squamous cell carcinoma; Adeno, adenocarcinoma; CRP, C-reactive protein; BM, bone marrow: SUV, standard uptake value; Ao, Aorta.

*Lymph node metastasis assessed by FDG-PET/CT (SUV max > 2.5 was considered positive for metastasis).

**Tumor diameter was measured three dimensionally based on T2-weighted MRI images.

In patients with cervical cancer, in the univariate Cox regression analysis, in addition to FIGO stage, tumor size, and hemoglobin level, BAR was found to be a significant indicator of PFS (Table 2). Survival analyses using the Kaplan–Meier method also demonstrated significantly worse PFS for patients in the higher-BAR group than those in the lower-BAR group (Fig. 1B(i)). In the multivariate analysis, in addition to decreased hemoglobin level (p = 0.014), higher BAR (p = 0.019) was significantly associated with shorter PFS (Table 2).

**Table 2:** Univariate and multivariate analyses for PFS in cervical cancer patients.

	Univariate Analysis		Multivariate Analysis	
	Hazard Ratio (95% CI)	p-value	Hazard Ratio (95% CI)	p-value
Age				
< 55	1	0.266	1	0.042
55 ≤	1.37 (0.78–2.45)		2.02 (1.02–4.12)	
Histology				
SCC	1	0.977	1	0.111
Adeno	1.06 (0.58–2.07)		1.81 (0.86–3.64)	
Tumor size				
< 40	1	0.001	1	0.078
40 ≤	2.54 (1.43–4.58)		1.88 (0.93–3.83)	
Hemoglobin (g/dl)				
< 10	1	0.001	1	0.010
10 ≤	0.34 (0.18–0.66)		0.35 (0.17–0.77)	
FIGO stage				
I-II	1	0.013	1	0.825
III-IV	2.11 (1.18–3.66)		1.08 (0.49–2.21)	
CRP (mg/l)				
< 1	1	0.875	1	0.332
1 ≤	0.95 (0.50–1.72)		0.71 (0.34–1.39)	
BM/Ao ratio				
< 1.5	1	< .0001	1	0.019
1.5 ≤	4.66 (2.27–8.76)		3.07 (1.21–7.21)	

CI, confidence interval; SCC, squamous cell carcinoma; Adeno, adenocarcinoma; FIGO, International Federation of Gynecology and Obstetrics; CRP, C-reactive protein; BM, Bone marrow; Ao, Aorta.

P-values were calculated using the two-sided Wald test in the Cox proportional hazard model.

In patients with ovarian and endometrial cancer, when all patients were analyzed, increased BAR was marginally correlated with shorter PFS (ovarian cancer : p = 0.058, endometrial cancer : p = 0.062; Supplemental Tables 9–10, Supplemental Fig. 4). However, when examined according to clinical stage, higher BAR was found to be associated with significantly shorter PFS only in advanced-stage patients (Fig. 1B(ii), (iii), Supplemental Fig. 4). Due to the limited number of patients exhibiting higher BAR, we did not perform multivariate in this patient population.

### Association between bone marrow FDG-uptake and the hematological parameters

To investigate the mechanism responsible for the increased BM FDG-uptake, the association between BAR and hematologic parameters was investigated. As shown, the correlations of BAR with neutrophil count were stronger than those with hemoglobin level, platelet count, red blood cell count, lymphocyte count, monocyte count, and eosinophil count (Fig. 1C, Supplemental Fig. 5). These results indicated that increased granulopoiesis is responsible, at least in part, for the increased BAR in patients with gynecological cancer.

### Mechanism responsible for increased BM FDG uptake

**Figure 2:**
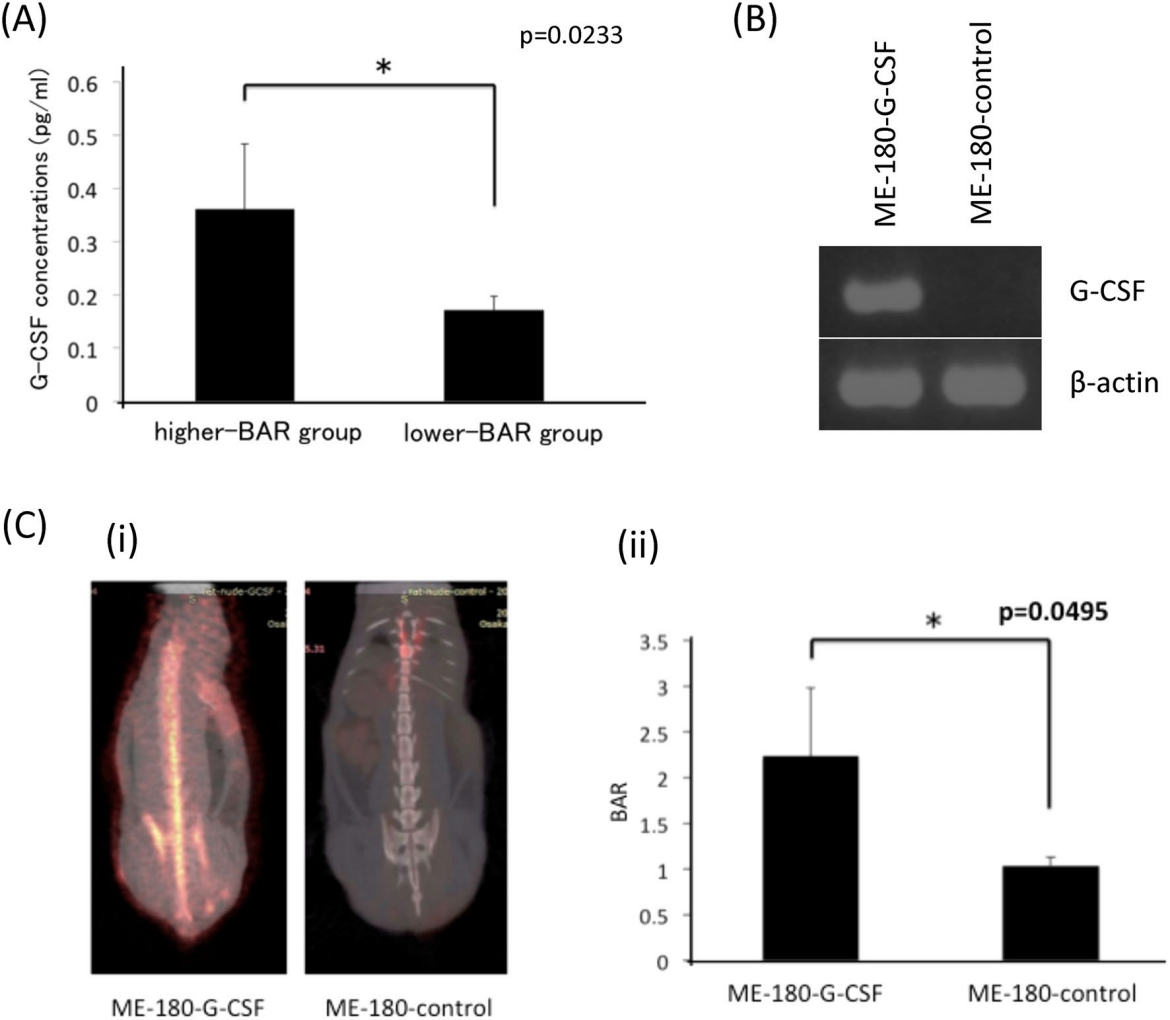
Tumor-derived G-CSF as a stimulator of BM FDG-uptake. (**A**) Correlation between and BAR and serum G-CSF levels in patients with cervical cancer. G-CSF concentrations in the serum of patients in higher and lower-BAR group of patients with cervical cancer were determined by ELISA. (**B**) Agarose gel electrophoresis of RT-PCR products for the expressions of G-CSF mRNA in ME-180-G-CSF or ME180-control cells. G-CSF and β-actin mRNA levels of ME-180 cells that had been incubated in the presence of 10% of FBS were assessed by RT-PCR. Full-length gels are presented in Supplemental Fig. 6A. (**C**) FDG-uptake in rat model of G-CSF-expressing cervical cancer. (i) Representative PET images obtained from rats bearing cervical cancer cells. (ii) The effect of tumor-derived G-CSF on the BM FDG-uptake in cervical cancer model rats.

To investigate the reason for the increased granulopoiesis in patients with increased BAR, we examined serum G-CSF levels using blood samples obtained. As shown (Fig. 2A), the serum G-CSF level was significantly higher in the higher-BAR-group than in the lower-BAR-group, which was consistent with G-CSF being a stimulator of BM metabolic activity^17^.

To directory demonstrate that tumor-derived G-CSF can increase BM FDG-uptake, we performed animal experiments. For this purpose, we performed FDG-PET/CT in a rat model of cervical or endometrial cancer, into which cervical or endometrial cancer cells stably transfected with G-CSF were inoculated. The expression of G-CSF in these cells were verified in vitro (Fig. 2B, Supplemental Fig. 6). As shown (Fig. 2C, Supplemental Fig. 6), subcutaneous inoculation of cancer cells stably transfected with a G-CSF-expressing vector (ME180-GCSF or Ishikawa-G-CSF) resulted in significantly increased BM FDG-uptake, which was in clear contrast to rats inoculated with ME180 cells stably transfected with a control vector (ME180-control or Ishikawa-control).

### Mechanism responsible for poor prognosis in patients with increased BM FDG-uptake

**Figure 3:**
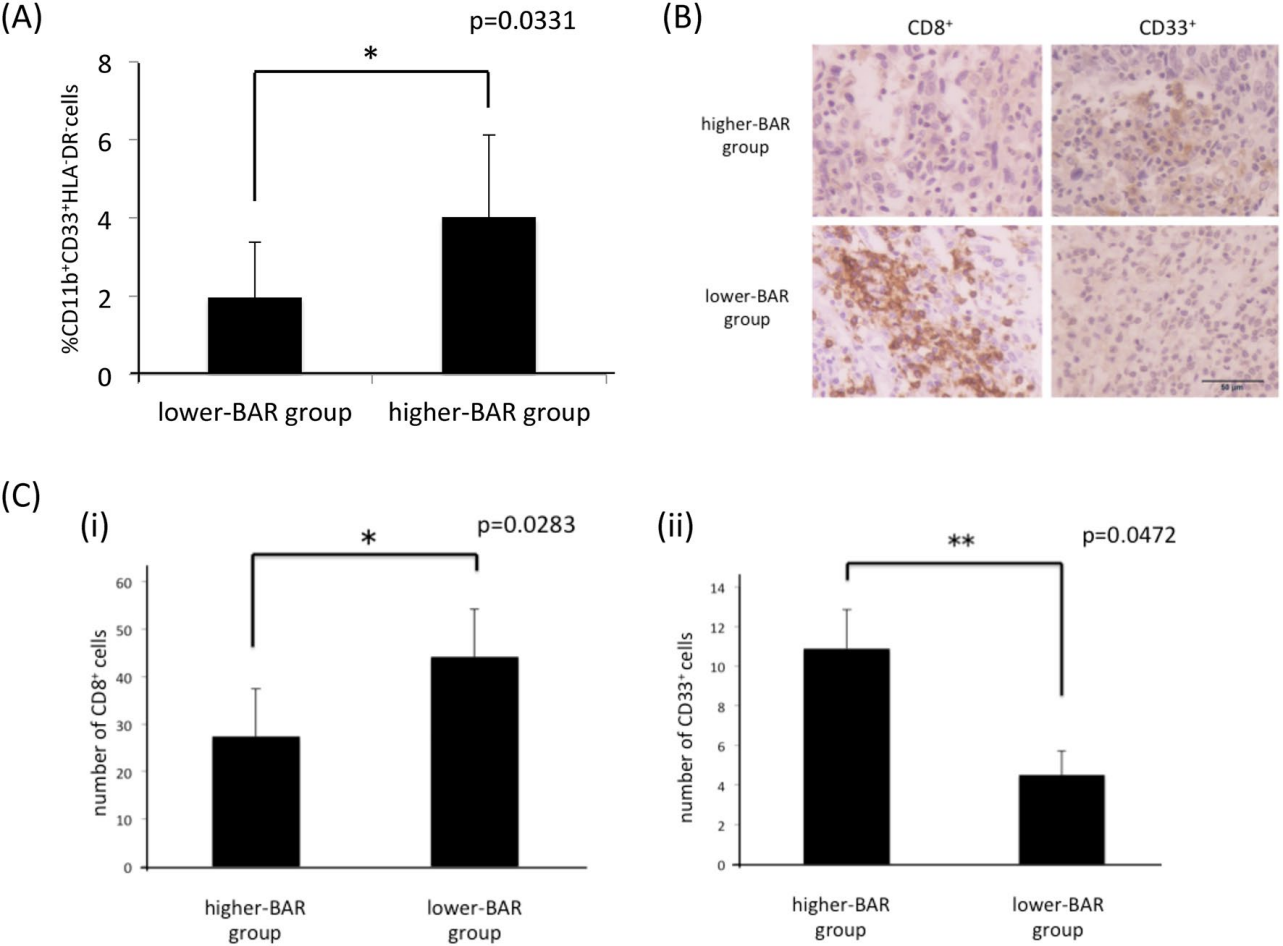
The association between BM FDG-uptake and MDSCs in patients with cervical cancer. (**A**) Circulating MDSC levels of patients with cervical cancer. Blood samples were obtained from the higher-BAR group (n = 5) and the lower-BAR group (n = 7). Human MDSCs, which were defined as CD11b^+^ CD33^+^ HLA^-^DR^-^ cells, were assessed using flow cytometry. Bars, SD. *p < 0.05, Wilcoxon rank sum test. (**B**-**C**), Immunoreactivities of cervical cancer for CD8 and CD33 according to BM FDG-uptake. Cervical tumor obtained from patients in the higher-BAR group (n = 5) and in the lower-BAR group (n = 7) were stained with anti-CD8 and anti-CD33 antibodies. (**B**) Representative images of primary tumors. (magnification: × 200). (**C**) Graphs depicting the number of CD8^+^ (i) and CD33^+^ cells. Tumor sections were observed using a bright field microscope (at × 200), and the number of CD8^+^ and CD33^+^ cells were counted. Then, the average value of 4 random fields is shown (Bars, SD, *p < 0.05, two-sided Student’s t test).

We reported previously that tumor-derived G-CSF induced MDSCs, and that MDSCs induced by tumor-derived G-CSF were responsible for the decreased survival in patients with cervical and endometrial cancer^14,18–22^. To investigate the mechanism responsible for the poor prognosis of patients in the higher-BAR group, we examined the number of MDSCs in peripheral blood and tumors of patients with gynecological cancer. As can be seen, significantly higher numbers of MDSCs, i.e. CD11b CD33^+^ HLA-DR-cells, were observed in patients in the higher-BAR group of patients with cervical cancer than patients in the lower-BAR group. (Fig. 3A). Consistent with this, in the immunohistochemical analyses, tumors obtained from patients in the higher-BAR group showed increased levels of CD33^+^ cells. Moreover, importantly, tumors obtained from patients in the higher-BAR group showed decreased CD8^+^ cells (Fig. 3B–C), which may indicate the suppressive activity of MDSCs.

**Figure 4:**
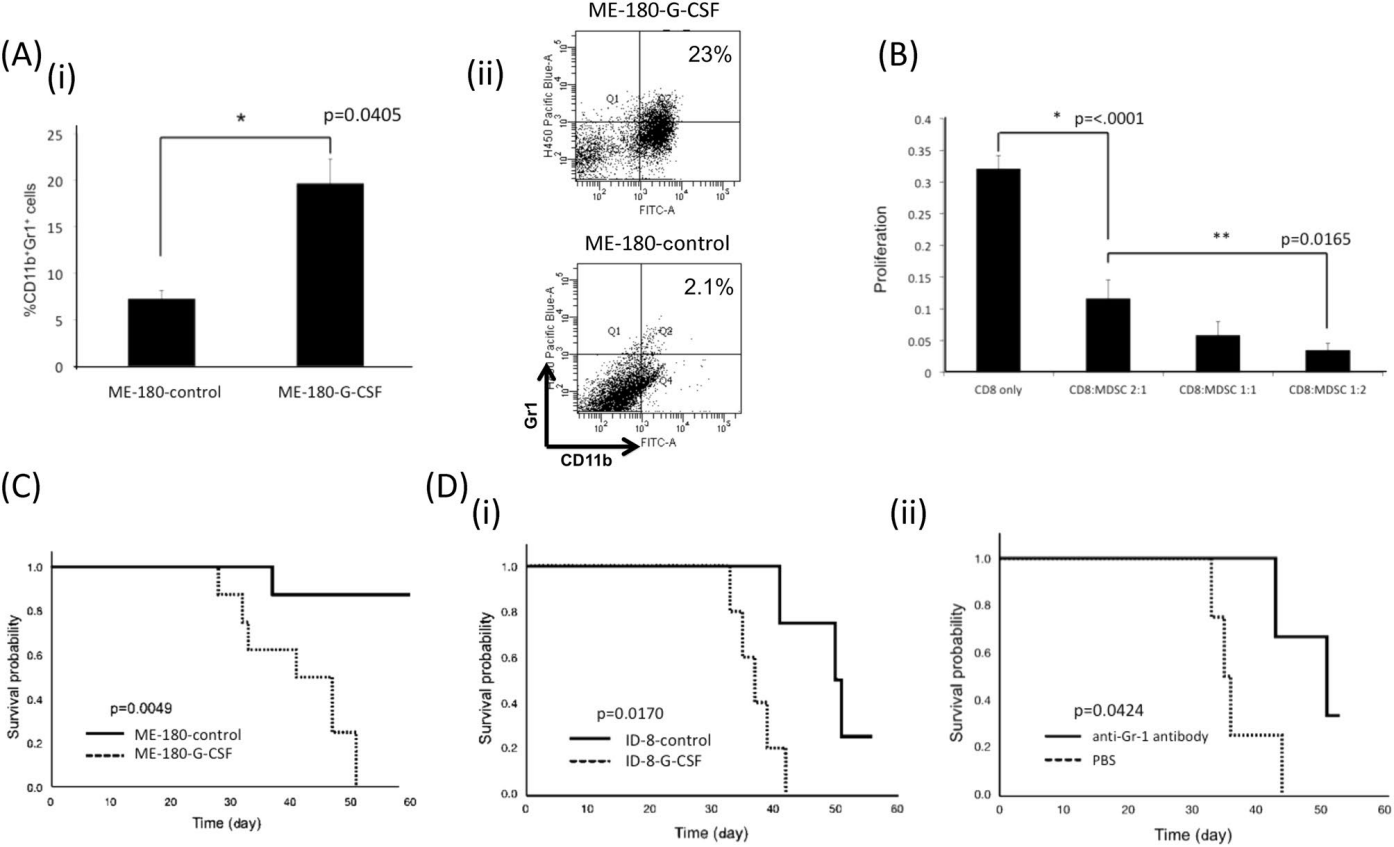
Roles of MDSCs and MDSC-mediated immune suppression in poor prognosis in patients exhibiting higher-BAR. (**A**) The effect of tumor-derived G-CSF on MDSC accumulation in subcutaneously inoculated tumors (mouse model of cervical cancer). ME180-G-CSF or ME180-control cells were subcutaneously inoculated into C57BL/6 mice (n = 6, for each group). Three weeks after the inoculation, their blood were collected for analyses. (i) CD11b^+^ Gr-1^+^ cell populations in tumors. Bars, SD. *p < 0.05, Two-sided Student t test. (ii) Representative dot plot. (**B**) Suppressive activity of MDSCs. CD11b^+^ Gr-1^+^ cells (MDSCs) were isolated from spleens of Balb/c mice subcutaneously inoculated with ME180-G-CSF. CD8^+^ T cells (2 × 10^5^ cells/well) were isolated from syngeneic mice and co-cultured with MDSCs at the indicated ratios. Cells were incubated for 72 h, after which BrdU was added for an additional 24 h. T cell proliferation was determined by BrdU incorporation. Bars, SD. *p < 0.05, Two-sided Student’s t test. (**C**) Impact of tumor-derived G-CSF on the survival of mice bearing cervical cancer. ME180-G-CSF or ME180-control cells were subcutaneously inoculated into C57BL/6 mice (n = 6, for each group). Then, the survival rate was estimated using Kaplan–Meier method. (**D**) Impact of tumor-derived G-CSF and MDSC on the survival of ovarian cancer-bearing mice. (i) Impact of tumor-derived G-CSF on the survival of ovarian cancer-bearing mice assessed using the Kaplan–Meier method. (ii) Effects of MDSC-inhibition using an anti-Gr-1 antibody on the survival of G-CSF-expressing ovarian cancer. Immunocompetent C57BL/6 mice were subcutaneously inoculated with 1.0 × 10^6^ ID-8 cells/mouse. Fourteen days after inoculation, mice were randomized into 2 groups that were administered either Gr-1 antibody (n = 5) or PBS (n = 5), every other day (day 14, 16, 18, 20). Kaplan–Meier estimates of survival is shown.

To directly demonstrate that immune suppression induced by MDSCs is responsible for the poor prognosis in patients in the higher-BAR group, we performed in vivo and in vitro experiments. As shown, ME180-G-CSF-derived tumor-bearing mice displayed markedly increased MDSC frequencies in their blood and tumors compared with the ME-180-control-derived tumor-bearing mice (Fig. 4A, Supplemental Fig. 7). Of note, MDSCs (CD11b^+^ Gr-1^+^ cells) isolated from mice significantly inhibited T cell proliferation (Fig. 4B), which was consistent with the immunosuppressive nature of MDSCs. Moreover, in the survival analysis, ME-180-G-CSF-derived cervical cancer-bearing mice showed significantly shorter survival than that ME-180-control-derived tumor-bearing mice (Fig. 4C OS: 42 vs. 60 days, p = 0.0049), which was consistent with the results from our recent report showing decreased survival in mice bearing G-CSF-expressing endometrial cancer^19^.

To overcome the weakness of animal studies using nude mice, we tried to use immunocompetent mice carrying mouse cancer cells. Because mouse cervical cancer or endometrial cancer cell lines were not available, we employed mice ovarian cancer cells, ID-8. Using this cell line, we first established G-CSF-expressing ovarian cancer cells that had been stably transfected with G-CSF (ID-8-G-CSF). The expression levels of G-CSF in cells were verified in vivo (Supplemental Fig. 6). As shown, the survival in ID-8-GCSF-derived tumor-bearing mice was shorter than that in ID-8-control-derived tumor-bearing mice (Fig. 4D(i) OS: 37 vs. 50.5 days, p = 0.0170). When ID-8-GCSF-derived tumor-bearing mice were treated with an anti-Gr-1 antibody, the survival was significantly prolonged (Fig. 4D(ii) OS: 38 vs. 44 days, p = 0.0424).

## Discussion

In the current study, we have found that increased BM FDG-uptake, as represented by increased BAR, is observed in roughly 10% of gynecological cancer patients and is associated with shorter survival.

In the area of gynecological cancer, 2 previous studies have investigated the prognostic significance of BM FDG-uptake^23,24^: both studies included patients with cervical cancer and their conclusions were similar to ours, but performed no mechanistic investigations. For ovarian and endometrial cancer, our study is the first investigate the prognostic significance of BM FDG uptake. As shown (Fig. 1B), BM FDG uptake provided prognostic information only in advanced-stage disease. Similarly, in cervical cancer, the prognostic significance of BM FDG uptake was greater in advanced-stage patients than in in early-stage patients (data not shown). The reason for this, as well as whether this finding is universal, remains unknown. However, previous investigations have suggested that pretreatment neutrophilia as well as the greater tumor G-CSF expression are more frequently observed in advanced-stage disease than in early-stage disease in endometrial or ovarian cancer patients^22–25^. These results may indicate that the amount of tumor-derived G-CSF is greater in advanced-stage disease than in early-stage disease. Thus, there is a possibility that the impact of MDSC-mediated immune suppression in tumor microenvironment is greater in advanced-stage disease than in early-stage disease. Future validation studies are required including larger numbers of patients.

To our knowledge, our study is the only that investigated the causative mechanism responsible for the clinical findings. Previous investigations have suggested that BM FDG-uptake can be affected by glucose metabolism in erythroid cells in the BM^6^. However, in our study, RBC count and hemoglobin level showed weaker correlations with BM FDG-uptake than WBCs or neutrophils (Fig. 1C), indicating that increased granulopoiesis plays a greater role in BM FDG-uptake than erythropoiesis in patients with gynecological cancer. Consistent with this, in the mechanistic investigations, we demonstrated that tumor-derived G-CSF is responsible for the increased metabolic activity of BM. We also showed that gynecological cancers developed in patients or mice with increased BM FDG-uptake frequently accompany leukocytosis or neutrophilia, but show immunosuppressive tumor microenvironment represented by increased MDSCs and decreased CD8^+^ T cells. These results indicated that immune suppression mediated by G-CSF-induced MDSCs may be involved in the mechanism responsible for the poor prognosis of patients with increased BM FDG-uptake.

This study may have important clinical implications. As shown in Supplemental Fig. 8, although FDG-uptake of the primary tumor is a significant prognosticator in cervical cancer patients, it provided no prognostic information in endometrial or ovarian cancer patients. These results may indicate that BM FDG-uptake is superior to FDG-uptake of the primary tumor as a prognosticator in gynecological cancer patients. Thus, by evaluating the BM FDG-uptake, it may be possible to identify patients who are at high risk of progression after current standard gynecological cancer treatments. Considering the high probability of treatment failure, careful post-treatment follow-up can be recommended for patients exhibiting increased BM FDG-uptake. Our mechanistic investigations suggested that treatment targeting MDSCs may have therapeutic efficacy in this patient population (Fig. 4D(ii)). Currently, no specific inhibitor of human MDSCs has been identified. However, we believe that the combination of conventional treatments (i.e., chemotherapy) with MDSC-targeting agents will improve the prognosis of patients with gynecological cancer exhibiting increased BM FDG-uptake.

The current study may open further research opportunities. Although our mechanistic investigations focused on the “tumor-derived G-CSF” and G-CSF-induced MDSCs, other tumor-derived factors may also play roles in the increased BM FDG-uptake in a G-CSF-independent manner. Moreover, we cannot exclude the possibility that other stromal cells in the tumor microenvironment might be stimulated by tumor-derived G-CSF to create premetastatic niche. Accordingly, the mechanism responsible for the increased BM FDG-uptake should be investigated further.

The limitations of our study need to be addressed. The first is that our clinical study was conducted at a single institution including a relatively small number of patients. Second, we did not evaluate the patient diabetic status or serum albumin, which may affect BM metabolic activity, because the information is not available for all patients. Third, we employed BAR to evaluate BM FDG-uptake. In a previous study conducted by Jeong et al., BM-to-liver uptake ratio of FDG-uptake^22^. A more recent study conducted by Seban et al., BM SUVmax was employed to evaluate the prognostic significance of BM FDG-uptake^23^. However, in our patient population, BM SUVmax did not provide prognostic information. Moreover, which vertebrae are the best representative of bone marrow activity remains unknown. In the current study, to measure FDG-uptake of the BM, an ROI was drawn over the body of each of 5 vertebrae (Th8–Th12), based on the previous studies that selected a lower thoracic vertebral bodies to represent the bone marrow activity^26,27^. Thus, further research is needed to develop an optimal approach to evaluate BM FDG-uptake in patients with cancer. The fourth is our experimental design. In animal studies, we used three gynecological cancer models; cervical, endometrial and ovarian cancers. It would be better using single animal model in all experiments to investigate the specific mechanism.

In conclusion, we have found that increased BM FDG-uptake is an indicator of poor prognosis in patients with gynecological cancer. We also showed that tumor-derived G-CSF is responsible for the increased BM metabolic activity, and that immune suppression mediated by G-CSF-induced MDSCs may be involved in the poor prognosis of patients with increased BM FDG-uptake. Future validation studies including larger number of patients need to be conducted.

## Supplementary Information

Supplementary Information 1.

Supplementary Information 2.
