# Supplementary material for: Prognostic significance of bone marrow FDG uptake in patients with gynecological cancer
Source: Sci Rep. 2021 Jan 26;11:2257. doi: 10.1038/s41598-021-81298-1 (PMC7838412; doi:10.1038/s41598-021-81298-1)
Supplement: Supplementary file 1 — Supplementary Information 1. [file 41598_2021_81298_MOESM1_ESM.docx]

**Supplemental Figure legends**

**Supplemental Figure 1.** A region of interest (ROI) placed over the intra-aorta-area (orange allow) and the vertebral body of 5 vertebrae (Th8–Th12, blue allow).

**Supplemental Figure 2.** Receiver operating characteristic curves for (A) maximum standardized uptake value (SUVmax) and (B) BM-to-aorta uptake ratio (BAR) for predicting progression.

**Supplemental Figure 3.** BM FDG-uptake in patients with gynecologic cancer. Kaplan–Meier estimates of progression-free survival in (A) patients with cervical cancer (higher-BM SUVmax group vs. lower-BM SUVmax group; n=231) (B) patients with endometrial cancer (higher-BM SUVmax group vs. lower-BM SUVmax group; n=161) and (C) patients with ovarian cancer (higher-BM SUVmax group vs. lower- BM SUVmax group; n=167).

**Supplemental Figure 4.** BAR in patients with gynecologic cancer. (A) Kaplan–Meier estimates of progression-free survival in (i) patients with early stage ovarian cancer (higher-BAR group vs. lower-BAR group; n=80) and (ii) all patients with ovarian cancer (higher-BAR group vs. lower-BAR group; n=167) (B) Kaplan–Meier estimates of progression-free survival in (i) patients with early stage endometrial cancer (higher-BAR group vs. lower-BAR group; n=48) and (ii) all patients with endometrial cancer (higher-BAR group vs. lower-BAR group; n=161).

**Supplemental Figure 5.** Correlation between BAR with hematologic parameters in patients with cervical cancer. (A) Hemoglobin level, (B) platelets, (C) RBC count, (D) lymphocytes, (E) monocytes, (F) eosinophils, and (G) basophils. Spearman correlation coefficient was used for analysis. The black circle; a density ellipse indicating correlation with a 95% confidence interval.

**Supplemental Figure 6.** (A) Full-length agarose gel electrophoresis of RT-PCR products for the expressions of G-CSF mRNA in ME-180-G-CSF, ME-180-control, ID-8-G-CSF, ID-8-control, Ishikawa-G-CSF or Ishikawa-control cells. G-CSF and β-actin mRNA levels of cells that had been incubated in the presence of 10% of FBS were assessed by RT-PCR. (B) The effect of tumor-derived G-CSF on the BM FDG-uptake in endometrial cancer model rats.

**Supplemental Figure 7.** The effect of tumor-derived G-CSF on MDSC accumulation in subcutaneously inoculated tumors (mouse model of cervical cancer). ME180-G-CSF or ME180-control cells were subcutaneously inoculated into C57BL/6 mice (n=6, for each group). Three weeks after the inoculation, their tumor cells were collected for analyses. (i) CD11b^+^ Gr-1^+^ cell populations in tumors. Bars, SD. *p < 0.05, Two-sided Student t test. (ii) Representative dot plot.

**Supplemental Figure 8.**

The prognostic significance of FDG-uptake of the primary tumor. A cut-off value of SUVmax of the primary tumor for predicting progression was determined using ROC analysis, and the optimal cut-off value was defined as 8.6. (A) Kaplan–Meier estimates of progression-free survival in cervical cancer patients. (i) all patients [higher-FDG uptake group (n=119) vs. lower-FDG uptake group (n=112)], (ii) early stage [higher-FDG uptake group (n=89) vs. lower-FDG uptake group (n=86)], (iii) advanced stage cervical cancer[higher-FDG uptake group (n=22) vs. lower-FDG uptake group (n=34)]. (B) Kaplan–Meier estimates of progression-free survival in ovarian cancer patients. (i) all patients [higher-FDG uptake group (n=67) vs. lower-FDG uptake group (n=100)], (ii) early stage [higher-FDG uptake group (n=24) vs. lower-FDG uptake group (n=56)], (iii) advanced stage cervical cancer[higher-FDG uptake group (n=43) vs. lower-FDG uptake group (n=44)]. (C) Kaplan–Meier estimates of progression-free survival in endmetrial cancer patients. (i) all patients [higher-FDG uptake group (n=77) vs. lower-FDG uptake group (n=84)], (ii) early stage [higher-FDG uptake group (n=24) vs. lower-FDG uptake group (n=24)], (iii) advanced stage cervical cancer[higher-FDG uptake group (n=55) vs. lower-FDG uptake group (n=60)].
