# Supplementary material for: Prognostic significance of bone marrow FDG uptake in patients with gynecological cancer
Source: Sci Rep. 2021 Jan 26;11:2257. doi: 10.1038/s41598-021-81298-1 (PMC7838412; doi:10.1038/s41598-021-81298-1)
Supplement: Supplementary file 2 — Supplementary Information 2. [file 41598_2021_81298_MOESM2_ESM.pdf]

Supplementary Information

Prognostic significance of bone marrow FDG uptake in patients with gynecological cancer

Kotaro Shimura[1,5], Seiji Mabuchi[1,2,5], Naoko Komura[1,5], Eriko Yokoi[1], Katsumi Kozasa[1], Tomoyuki Sasano[3], Mahiru Kawano[1], Yuri Matsumoto[1], Tadashi Watabe[4], Michiko Kodama[1], Kae Hashimoto[1], Kenjiro Sawada[1], Jun Hatazawa[4], and Tadashi Kimura[1]

[1] Department of Obstetrics and Gynecology, Osaka University Graduate School of Medicine. 2-2 Yamadaoka, Suita, Osaka, 565-0871 Japan.
[2] Department of Obstetrics and Gynecology, Nara Medical University, Kashihara, Nara 634-8522, Japan.
[3] Department of Gynecologic Oncology and Reproductive Medicine, The University of Texas MD Anderson Cancer Center
[4] Department of Nuclear Medicine and Tracer Kinetics, Osaka University Graduate School of Medicine. 2-2 Yamadaoka, Suita, Osaka, 565-0871 Japan.
[5] These authors equally contributed to this work.

- **Correspondence to:** Seiji Mabuchi, M.D., Ph.D. (smabuchi@gyne.med.osaka-u.ac.jp)

Supplementary Information

Supplementary Figure 1-8

Supplementary table 1-10

Supplemental figure 1

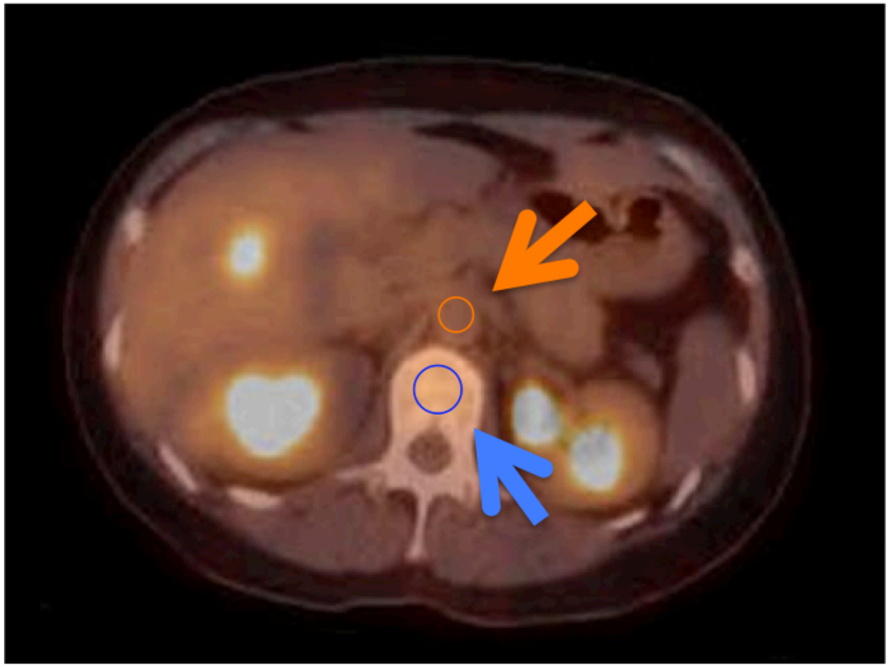

Supplemental figure 2

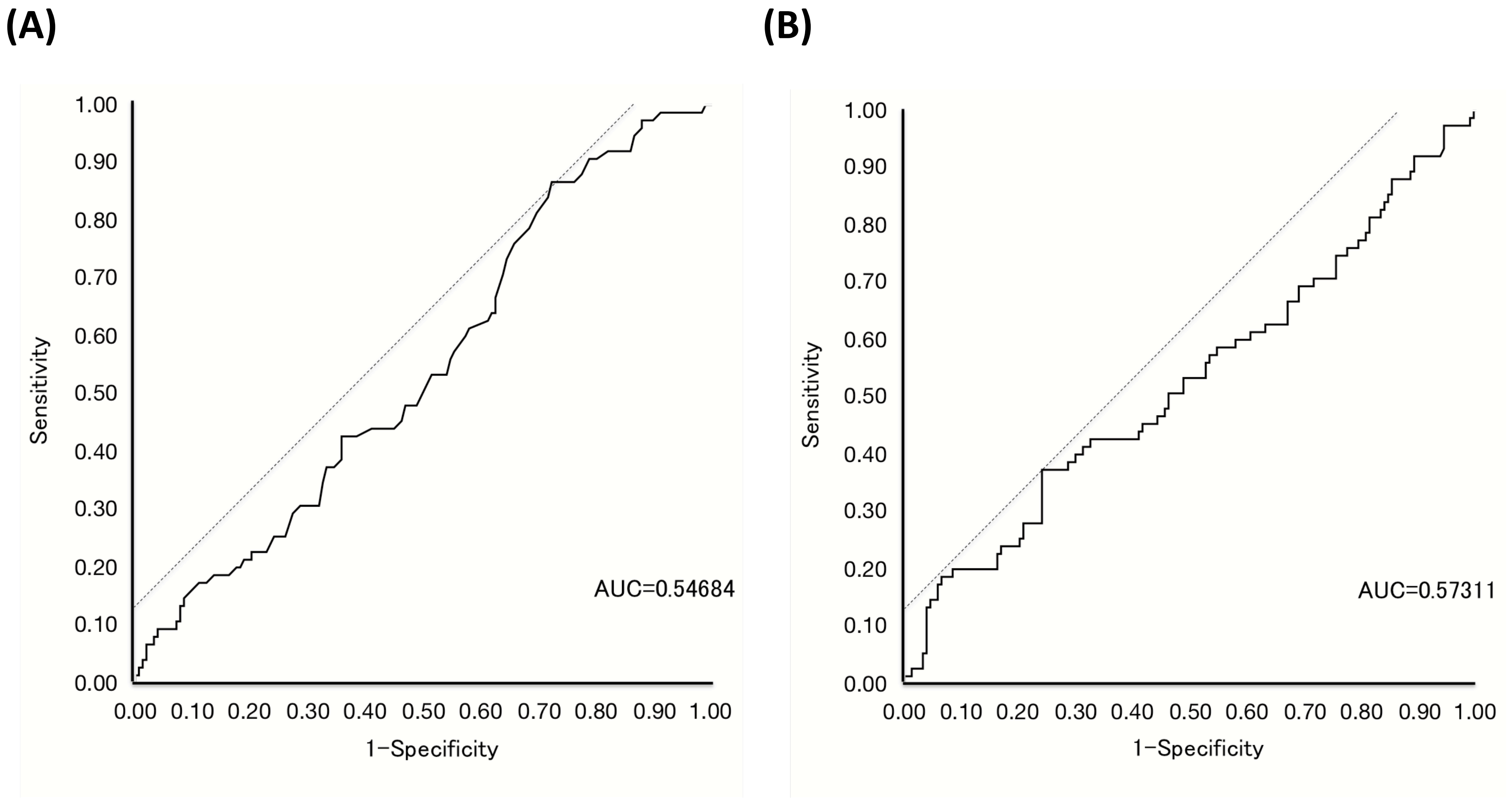

Supplemental figure 3

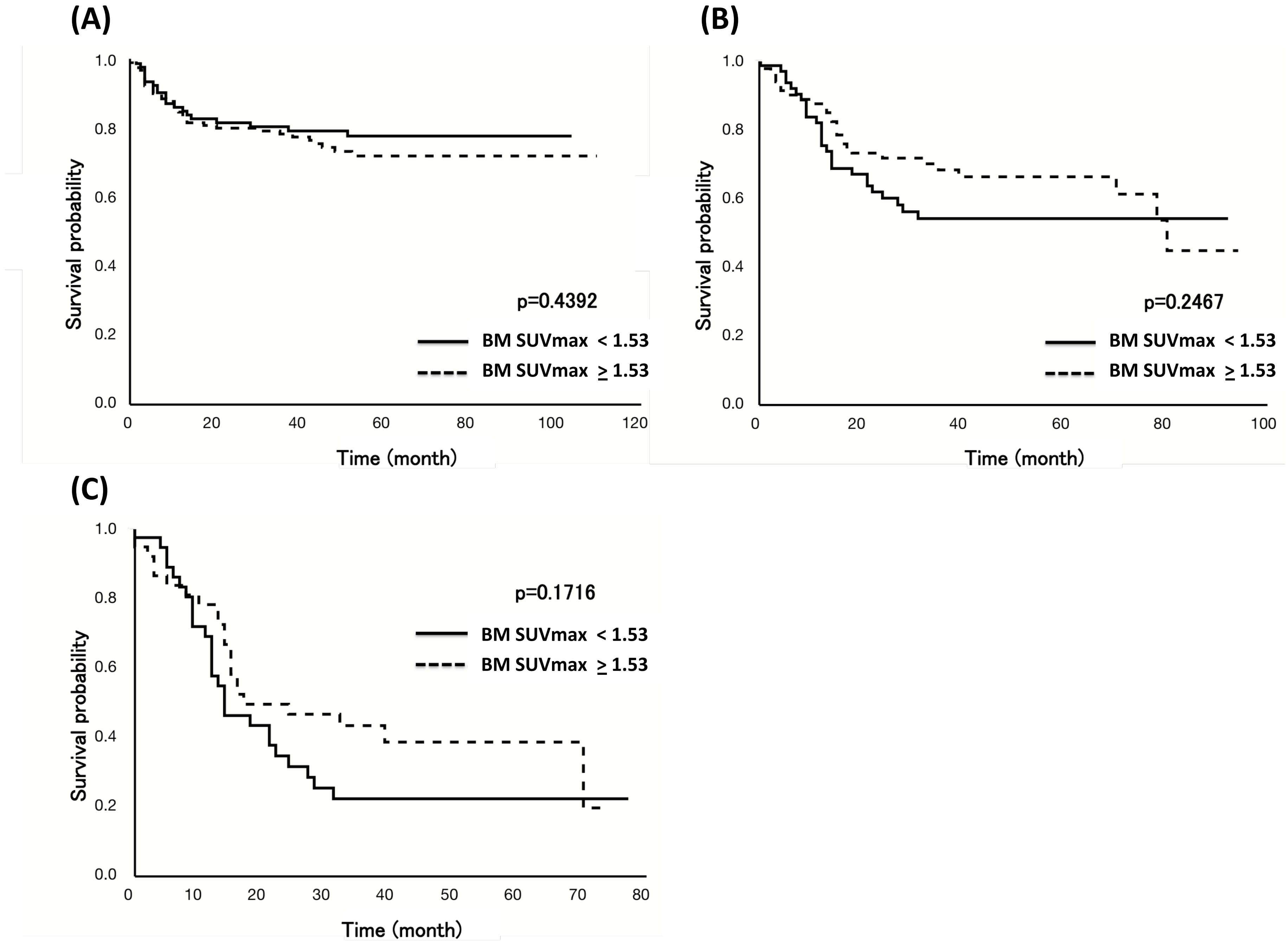

Supplemental figure 4

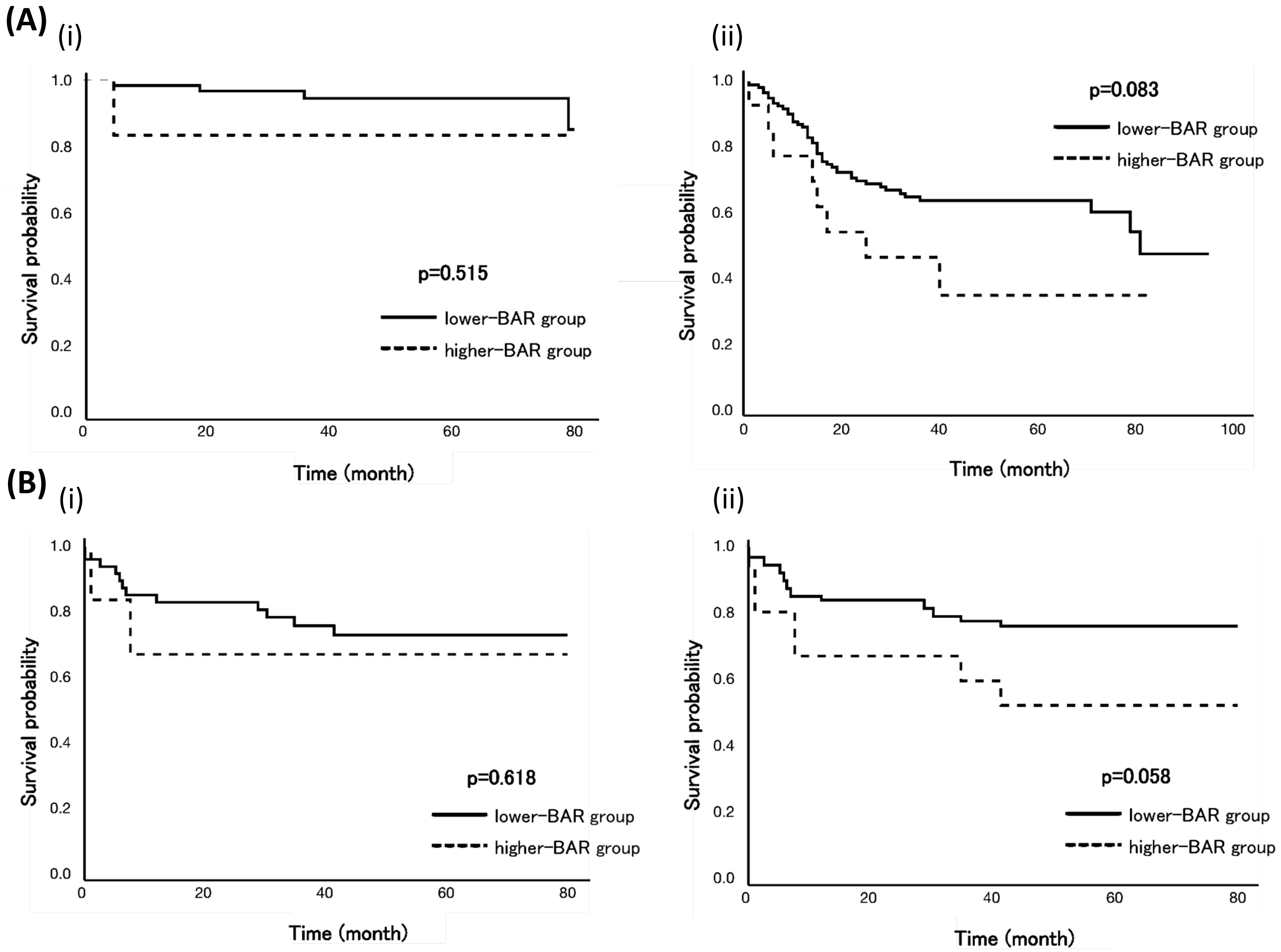

Supplemental figure 5

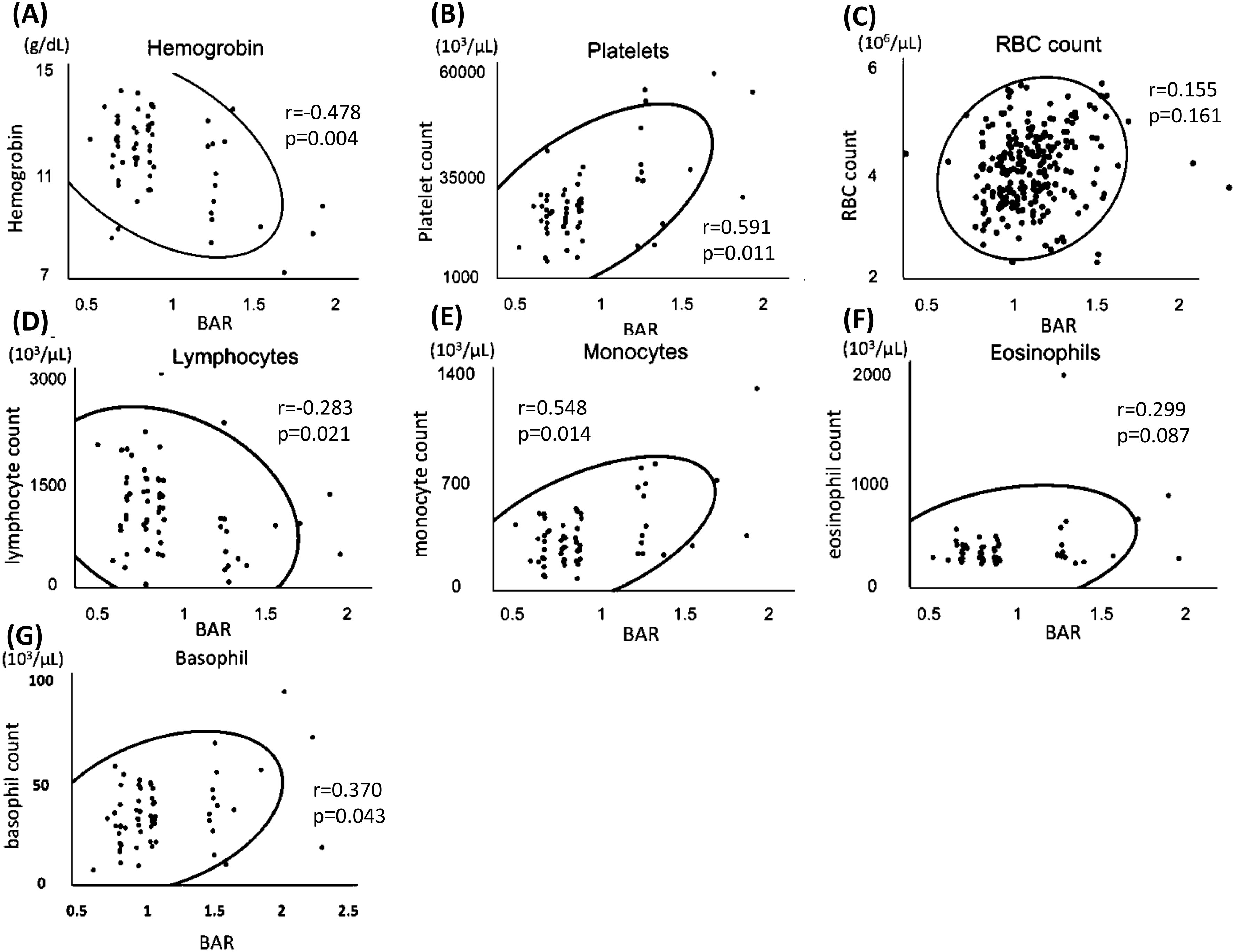

Supplemental figure 6

(A)

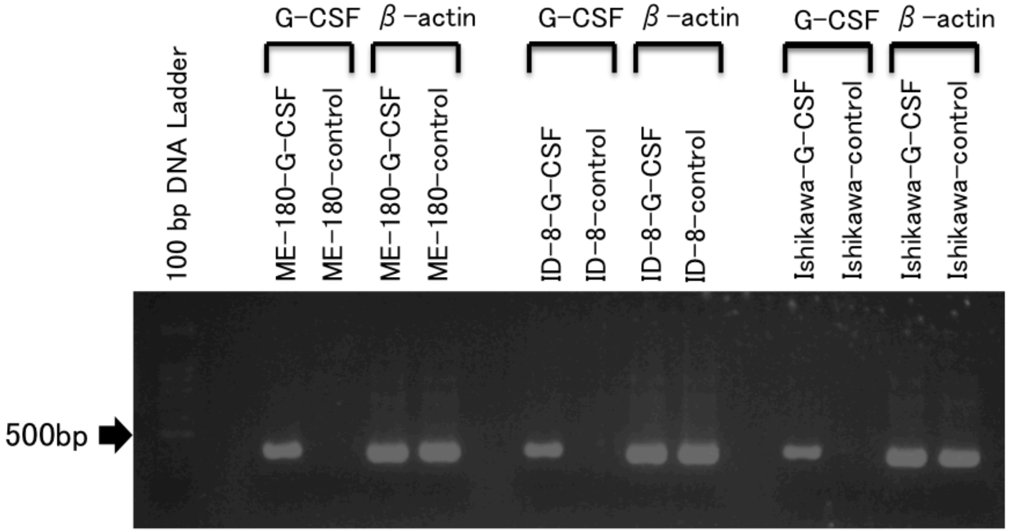

(B)

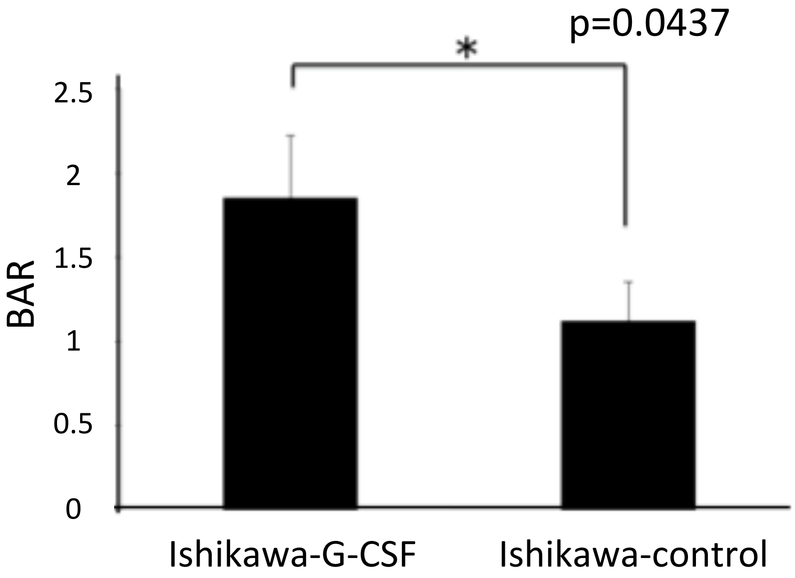

Supplemental figure 7

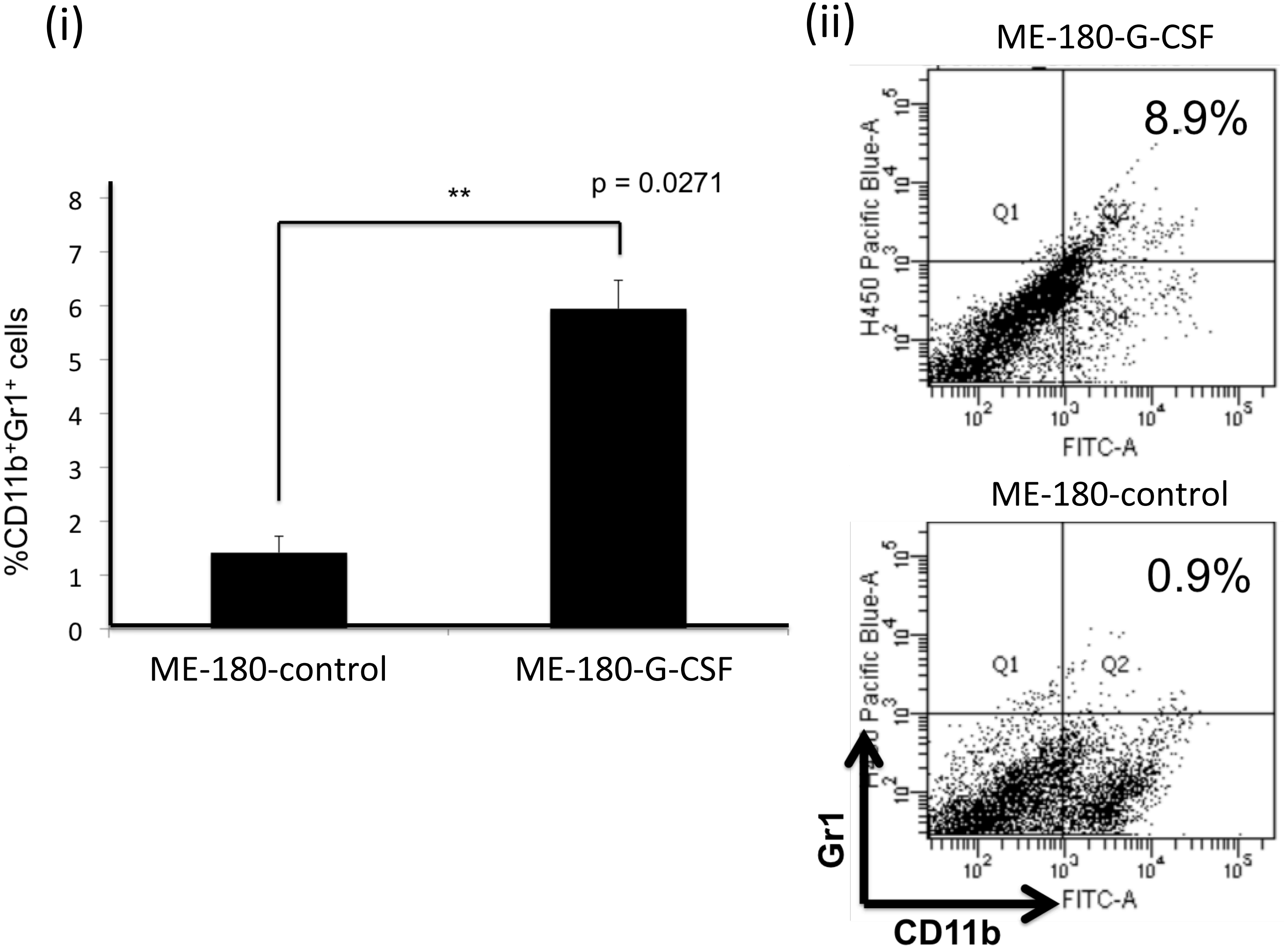

Supplemental figure 8

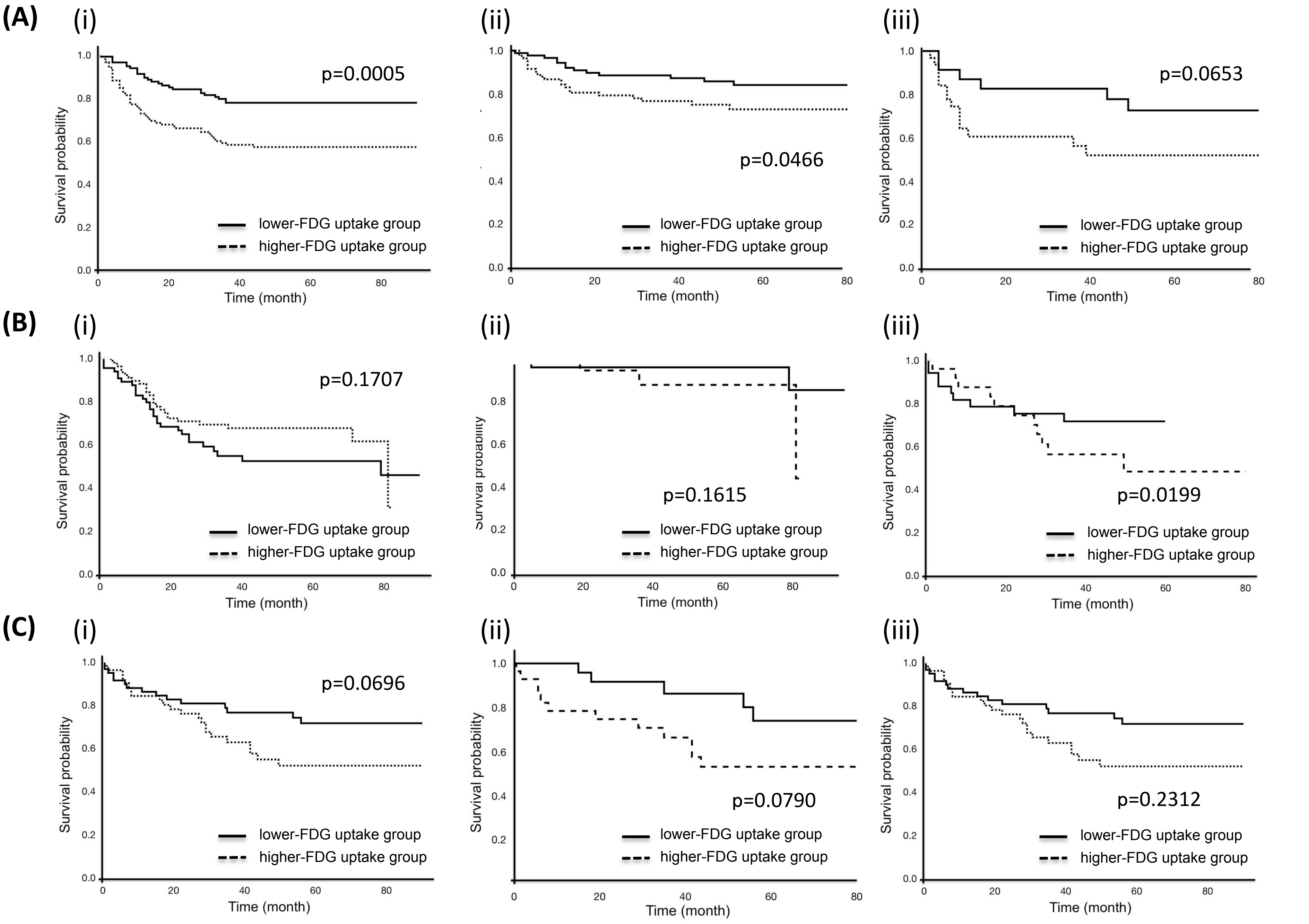

Supplemental Table 1. Clinical characteristics of the 231 cervical cancer patients

		No. of Patients (%) Median (Range)	BM SUVmax ≤1.5 (n=99)	BM SUVmax >1.5 (n=132)	p-value
Age (y.o.)		54 (26-96)	51 (28-96)	56 (26-88)	0.512
FIGO stage	I	100 (43)	44 (44)	56 (42)	0.123
	II	75 (33)	32 (32)	43 (33)	
	III	26 (11)	4 (4)	22 (17)	
	IV	30 (13)	19 (19)	11 (8)	
Primary treatment	Surgery	119 (51)	48 (48)	71 (55)	0.721
	Radiotherapy	110 (48)	51 (51)	59 (44)	
	Others	2 (1)	1 (1)	1 (1)	
Histology	SCC	161 (70)	88 (89)	73 (55)	0.067
	Adeno	57 (25)	8 (8)	49 (37)	
	Others	13 (5)	3 (3)	10 (8)	
Lymph node metastasis*	Positive	47 (20)	22 (22)	25 (19)	0.691
	Negative	184 (80)	77 (78)	107 (81)	
Tumor size (cm) **		35 (5-100)	45 (5-100)	35 (5-88)	0.171
Hemoglobin (g/dl)		12.6 (7.2-15.3)	8.4 (7.3-12.7)	12.1 (8.2-15.3)	0.137
CRP (mg/l)		0.04 (0-18.1)	0.21 (0-18.1)	0.04 (0-13.6)	0.141
BM SUVmax		1.6 (0.56-2.8)	1.13 (0.56-1.53)	1.82 (1.55-2.8)	<.0001

BM, bone marrow; SUV, standard uptake value; FIGO, International Federation of Gynecology and Obstetrics; SCC, squamous cell carcinoma; Adeno, adenocarcinoma ; CRP, C-reactive protein.

* Lymph node metastasis assessed by FDG-PET/CT (SUV max>2.5 was considered positive for metastasis).

** Tumor diameter was measured three dimensionally based on T2-weighted MRI images.

Supplemental Table 2. Clinical characteristics of the 161 endometrial cancer patients

Charactristics		No. of Patients (%) Median (Range)	BM SUVmax ≤1.53 (n=77)	BM SUVmax >1.53 (n=84)	p-value
Age (y.o.)		58 (23-89)	59 (23-89)	53 (27-83)	0.717
FIGO stage	I	27 (16)	15 (19)	12 (14)	0.031
	II	21 (13)	16 (21)	5 (6)	
	III	54 (34)	20 (26)	34 (40)	
	IV	59 (37)	26 (34)	33 (39)	
Primary treatment	Surgery	33 (20)	19 (25)	14 (17)	0.066
	Surgery+Chemotherapy	112 (70)	49 (64)	63 (75)	
	Chemotherapy	13 (8)	9 (12)	4 (4)	
	Others	3 (2)	0 (0)	3 (4)	
Histology	Endmetrioid	130 (81)	66 (86)	64 (71)	0.582
	Non-endometrioid	31 (19)	11 (14)	20 (29)	
Lymph node metastasis*	Positive	49 (30)	22 (29)	27 (32)	0.814
	Negative	112 (70)	55 (71)	57 (68)	
Tumor size (cm) **		15 (0.5-88)	14 (0.5-72)	17 (1-88)	0.112
Hemoglobin (g/dl)		11.7 (6.3-14.4)	13.1 (8.8-14.4)	8.4 (6.3-10.7)	0.029
CRP (mg/l)		0.04 (0-8.53)	0.033 (0-4.14)	0.41 (0-8.53)	0.313

BM, bone marrow; SUV, standard uptake value; FIGO, International Federation of Gynecology and Obstetrics; CRP, C-reactive protein.

* Lymph node metastasis assessed by FDG-PET/CT (SUV max>2.5 was considered positive for metastasis).

** Tumor diameter was measured three dimensionally based on T2-weighted MRI images.

Supplemental Table 3. Clinical characteristics of the 167 ovarian cancer patients

		No. of Patients (%)<br>Median (Range)	BM SUVmax ≤1.53<br>(n=85)	BM SUVmax >1.53<br>(n=82)	p-value
Age (y.o.)		57 (19-81)	53 (22-81)	59 (19-78)	0.811
FIGO stage	I	67 (40.1)	30 (35)	37 (45)	0.652
	II	13 (7.8)	6 (7)	7 (9)	
	III	50 (29.9)	29 (34)	21 (26)	
	IV	37 (22.2)	20 (24)	17 (21)	
Primary treatment	Surgery	18 (11)	14 (17)	4(5)	0.173
	Surgery + Chemotherapy	147 (88)	70 (82)	77 (94)	
	Others	2 (1)	1 (1)	1 (1)	
Histology	Serous	88 (53)	51 (60)	37 (45)	0.107
	Mucinous	16 (10)	10 (12)	6 (7)	
	Clear cell	35 (21)	14 (16)	21 (26)	
	Endometrioid	27 (16)	10 (12)	17 (21)	
Lymph node metastasis*	Positive	44 (26)	31 (25)	13 (38)	0.047
	Negative	123 (74)	54 (75)	69 (62)	
Tumor size (cm) **		9.8 (0.6-27)	9.0 (0.6-27)	12.1 (1.7-25)	0.399
Hemoglobin (g/dl)		11.1 (6.9-14.3)	13.1 (6.9-14.3)	9.9 (7.2-11.2)	0.273
CRP (mg/l)		0.04 (0-14.3)	0.04 (0-11.6)	0.21 (0-14.3)	0.130
BM SUVmax		1.53 (1.08-2.36)	1.69 (1.08-1.53)	2.22 (1.55-2.8)	<.0001

BM, bone marrow; SUV, standard uptake value; FIGO, International Federation of Gynecology and Obstetrics; CRP, C-reactive protein.
* Lymph node metastasis assessed by FDG-PET/CT (SUV max>2.5 was considered positive for metastasis).
** Tumor diameter was measured three dimensionally based on T2-weighted MRI images.

Supplemental Table 4. Univariate and multivariate analyses for PFS in cervical cancer patients

		Univariate Analysis		Multivariate Analysis	
		Hazard Ratio (95% CI)	p-value	Hazard Ratio (95% CI)	p-value
Age	<55	1	0.266	1	0.064
	55≤	1.37 (0.78-2.45)		1.86 (0.96-3.67)	
Histology	SCC	1	0.843	1	0.119
	Adeno	1.06 (0.58-2.07)		1.78 (0.86-3.58)	
Tumor Size	<40	1	0.001	1	0.041
	40≤	2.54 (1.43-4.58)		2.03 (1.03-4.05)	
Hemoglobin (g/dl)	<10	1	0.001	1	0.004
	10≤	0.34 (0.18-0.66)		0.32 (0.16-0.68)	
FIGO stage	I-II	1	0.013	1	0.923
	III-IV	2.11 (1.18-3.66)		0.96 (0.44-1.94)	
CRP (mg/l)	<1	1	0.270	1	0.270
	1≤	0.95 (0.50-1.72)		0.68 (0.32-1.32)	
BM SUVmax	<1.53	1	0.440	1	0.472
	1.53≤	1.24 (0.72-2.19)		1.28 (0.65-2.61)	

CI, confidence interval; FIGO, International Federation of Gynecology and Obstetrics; CRP, C-reactive protein; BM, Bone marrow; SUV, standard uptake value.

P-values were calculated using the two-sided Wald test in the Cox proportional hazard model.

Supplemental Table 5. Univariate and multivariate analyses for PFS in ovarian cancer patients

		Univariate Analysis		Multivariate Analysis	
		Hazard Ratio (95% CI)	p-value	Hazard Ratio (95% CI)	p-value
Age	<55	1	0.416	1	0.268
	55≤	1.41 (0.25-1.81)		1.62 (0.89-2.37)	
Histology	Serous	1	0.276	1	0.411
	Non serous	1.06 (0.58-5.07)		1.05 (0.72-3.63)	
Tumor Size	<30	1	0.011	1	0.414
	30≤	2.07 (0.99-5.19)		1.76 (1.69-4.84)	
Hemoglobin (g/dl)	<10	1	0.001	1	0.032
	10≤	0.34 (0.13-1.04)		0.45 (0.11-1.73)	
FIGO stage	I-II	1	0.009	1	0.873
	III-IV	3.05 (1.01-4.11)		1.11 (0.44-2.12)	
CRP (mg/l)	<1	1	0.879	1	0.531
	1≤	0.98 (0.61-1.56)		0.88 (0.53-1.63)	
BM SUVmax	<1.53	1	0.253	1	0.312
	1.53≤	1.36 (0.80-2.32)		1.29 (0.17-3.92)	

CI, confidence interval; FIGO, International Federation of Gynecology and Obstetrics; CRP, C-reactive protein; BM, Bone marrow; SUV, standard uptake value.

P-values were calculated using the two-sided Wald test in the Cox proportional hazard model.

Supplemental Table 6. Univariate and multivariate analyses for PFS in endmetrial cancer patients

		Univariate Analysis		Multivariate Analysis	
		Hazard Ratio (95% CI)	p-value	Hazard Ratio (95% CI)	p-value
Age	<55	1	0.226	1	0.164
	55≤	1.47 (0.78-2.65)		1.86 (0.77-4.49)	
Histology	Endmetrioid	1	0.345	1	0.902
	Non-endometrioid	1.27 (0.57-2.17)		1.05 (0.43-2.46)	
Tumor Size	<40	1	0.013	1	0.002
	40≤	2.12 (1.03-3.58)		4.21 (1.70-10.38)	
Hemoglobin (g/dl)	<10	1	0.001	1	0.082
	10≤	0.34 (0.18-0.66)		0.36 (0.13-1.14)	
FIGO stage	I-II	1	0.021	1	0.437
	III-IV	2.31 (1.38-3.51)		2.10 (0.40-8.73)	
CRP (mg/l)	<1	1	0.593	1	0.187
	1≤	0.91 (0.40-1.54)		0.51 (0.16-1.35)	
BM SUVmax	<1.53	1	0.917	1	0.323
	1.53≤	1.14 (0.48-2.33)		1.61 (0.62-4.15)	

CI, confidence interval; FIGO, International Federation of Gynecology and Obstetrics; CRP, C-reactive protein; BM, Bone marrow; SUV, standard uptake value.

P-values were calculated using the two-sided Wald test in the Cox proportional hazard model.

Supplemental Table 7. Clinical characteristics of the lower- and higher-BAR group patients with endometrial cancer

Charactristics		lower-BAR group (n=140)	higher-BAR group (n=21)	p-value
Age (y.o.)		58 (23-89)	49 (29-78)	0.217
FIGO stage	I	23 (16)	4 (19)	0.717
	II	17 (12)	4 (19)	
	III	48 (34)	6 (29)	
	IV	52 (37)	7 (33)	
Primary treatment	Surgery	32 (23)	1 (5)	0.361
	Surgery+Chemotherapy	95 (68)	17 (81)	
	Chemotherapy	11 (8)	2 (10)	
	Others	2 (1)	1 (6)	
Histology	Endmetrioid	111 (79)	19 (90)	0.917
	Non-endometrioid	29 (21)	2 (10)	
Lymph node metastasis*	Positive	38 (27)	11 (52)	0.024
	Negative	102 (73)	10 (48)	
Tumor size (cm) **		15 (0.5-88)	13 (1-60)	0.412
Hemoglobin (g/dl)		12.1 (8.3-14.4)	9.4 (6.3-11.2)	0.037
CRP (mg/l)		0.048 (0-2.64)	0.28 (0-8.53)	0.083
BM SUV		1.54 (0.68-2,16)	2.04 (1.56-3.46)	0.025
BM/Ao ratio		1.05 (0.76-1.49)	1.9 (1.50-3.09)	<.0001

BAR, BM-to-Aorta ratio of FDG uptake; FIGO, International Federation of Gynecology and Obstetrics; CRP, C-reactive protein. BM, bone marrow; SUV, standard uptake value; Ao, Aorta.

* Lymph node metastasis assessed by FDG-PET/CT (SUV max>2.5 was considered positive for metastasis).

** Tumor diameter was measured three dimensionally based on T2-weighted MRI images.

Supplemental Table 8. Clinical characteristics of the lower- and higher-BAR group patients with ovarian cancer

		lower-BAR group (n=146)	higher-BAR group (n=21)	p-value
Age (y.o.)		57 (19-81)	61 (23-77)	0.782
FIGO stage	I	59 (42)	8 (38)	<.0001
	II	12 (9)	1 (5)	
	III	47 (34)	3 (14)	
	IV	28 (20)	9 (43)	
Primary treatment	Surgery	15 (10)	3 (14)	0.361
	Surgery + Chemotherapy	130 (89)	17 (81)	
	Others	1 (1)	1 (5)	
Histology	Serous	77 (53)	11 (52)	0.408
	Mucinous	13 (9)	3 (14)	
	Clear cell	28 (19)	7 (33)	
	Endometrioid	25 (17)	2 (10)	
Lymph node metastasis*	Positive	36 (25)	8 (38)	0.468
	Negative	110 (75)	13 (62)	
Tumor size (cm) **		9.8 (0.6-27)	11.4 (1.2-22)	0.377
Hemoglobin (g/dl)		11.7 (7.1-14.3)	8.9 (6.9-12.2)	0.293
CRP (mg/l)		0.04 (0-12.6)	0.41 (0-14.3)	0.130
BM SUV		1.69 (1.08-2,66)	2.22 (1.39-2.8)	0.007
BM/Ao ratio		1.02 (0.82-1.49)	1.53 (1.50-1.69)	<.0001

BAR, BM-to-Aorta ratio of FDG uptake; FIGO, International Federation of Gynecology and Obstetrics; CRP, C-reactive protein; BM, bone marrow; SUV, standard uptake value; Ao, Aorta.

* Lymph node metastasis assessed by FDG-PET/CT (SUV max>2.5 was considered positive for metastasis).

** Tumor diameter was measured three dimensionally based on T2-weighted MRI images.

Supplemental Table 9. Univariate analyses for PFS in ovarian cancer patients

		Univariate Analysis	
		Hazard Ratio (95% CI)	p-value
Age	<55	1	0.416
	55≤	1.41 (0.25-1.81)	
Histology	Serous	1	0.276
	Non serous	1.06 (0.58-5.07)	
Tumor Size	<30	1	0.011
	30≤	2.07 (0.99-5.19)	
Hemoglobin (g/dl)	<10	1	0.001
	10≤	0.34 (0.13-1.04)	
FIGO stage	I-II	1	0.009
	III-IV	3.05 (1.01-4.11)	
CRP (mg/l)	<1	1	0.879
	1≤	0.98 (0.61-1.56)	
BM/Ao Ratio	<1.5	1	0.083
	1.5≤	1.57 (0.88-2.12)	

CI, confidence interval; FIGO, International Federation of Gynecology and Obstetrics; CRP, C-reactive protein; BM, bone marrow; Ao, Aorta. P-values were calculated using the two-sided Wald test in the Cox proportional hazard model.

Supplemental Table 10. Univariate analyses for PFS in endmetrial cancer patients

		Univariate Analysis	
		Hazard Ratio (95% CI)	p-value
Age	<55	1	0.226
	55≤	1.47 (0.78-2.65)	
Histology	Endmetrioid	1	0.345
	Non-endometrioid	1.27 (0.57-2.17)	
Tumor Size	<40	1	0.013
	40≤	2.12 (1.03-3.58)	
Hemoglobin (g/dl)	<10	1	0.001
	10≤	0.34 (0.18-0.66)	
FIGO stage	I-II	1	0.021
	III-IV	2.31 (1.38-3.51)	
CRP (mg/l)	<1	1	0.593
	1≤	0.91 (0.40-1.54)	
BM/Ao Ratio	<1.5	1	0.058
	1.5≤	1.61 (1.07-2.36)	

CI, confidence interval; FIGO, International Federation of Gynecology and Obstetrics; CRP, C-reactive protein; BM, bone marrow; Ao, Aorta.
P-values were calculated using the two-sided Wald test in the Cox proportional hazard model.
